# Mosquito Small RNA Responses to West Nile and Insect-Specific Virus Infections in *Aedes* and *Culex* Mosquito Cells

**DOI:** 10.3390/v11030271

**Published:** 2019-03-18

**Authors:** Giel P. Göertz, Pascal Miesen, Gijs J. Overheul, Ronald P. van Rij, Monique M. van Oers, Gorben P. Pijlman

**Affiliations:** 1 Laboratory of Virology, Wageningen University & Research, Droevendaalsesteeg 1, 6708 PB Wageningen, The Netherlands; giel.goertz@wur.nl (G.P.G.); monique.vanoers@wur.nl (M.M.v.O.); 2 Department of Medical Microbiology, Radboud University Medical Center, Radboud Institute for Molecular Life Sciences, Geert Grooteplein Zuid 28, 6525 GA Nijmegen, The Netherlands; Pascal.Miesen@radboudumc.nl (P.M.); Gijs.overheul@radboudumc.nl (G.J.O.); Ronald.vanRij@radboudumc.nl (R.P.v.R.)

**Keywords:** small RNA, mosquito cells, RNAi, next-generation sequencing, *de novo* assembly, virus discovery, PIWI-interacting RNAs, small-interfering RNAs, West Nile virus, insect-specific viruses

## Abstract

Small RNA mediated responses are essential for antiviral defence in mosquitoes, however, they appear to differ per virus-vector combination. To further investigate the diversity of small RNA responses against viruses in mosquitoes, we applied a small RNA deep sequencing approach on five mosquito cell lines: *Culex tarsalis* CT cells, *Aedes albopictus* U4.4 and C6/36 cells, *Ae. aegypti* Aag2 cells (cleared from cell fusing agent virus and Culex Y virus (CYV) by repetitive dsRNA transfections) and *Ae. pseudoscutellaris* AP-61 cells. *De novo* assembly of small RNAs revealed the presence of Phasi Charoen-like virus (PCLV), Calbertado virus, Flock House virus and a novel narnavirus in CT cells, CYV in U4.4 cells, and PCLV in Aag2 cells, whereas no insect-specific viruses (ISVs) were detected in C6/36 and AP-61 cells. Next, we investigated the small RNA responses to the identified ISVs and to acute infection with the arthropod-borne West Nile virus (WNV). We demonstrate that AP-61 and C6/36 cells do not produce siRNAs to WNV infection, suggesting that AP-61, like C6/36, are Dicer-2 deficient. CT cells produced a strong siRNA response to the persistent ISVs and acute WNV infection. Interestingly, CT cells also produced viral PIWI-interacting (pi)RNAs to PCLV, but not to WNV or any of the other ISVs. In contrast, in U4.4 and Aag2 cells, WNV siRNAs, and pi-like RNAs without typical ping-pong piRNA signature were observed, while this signature was present in PCLV piRNAs in Aag2 cells. Together, our results demonstrate that mosquito small RNA responses are strongly dependent on both the mosquito cell type and/or the mosquito species and family of the infecting virus.

## 1. Introduction

Mosquitoes serve as primary vectors for the vast majority of arthropod-borne (arbo)viruses, which pose a global health threat to humans and other vertebrates. With the introduction of next-generation sequencing technologies and metagenomics into the field of virology, it becomes increasingly clear that many insects and insect cell lines, including mosquitoes, carry persistently infecting insect-specific viruses (ISVs) [1,2,3,4]. The presence of ISVs in mosquitoes and mosquito cell lines can interfere with the infection and replication of arboviruses [5,6,7,8,9,10,11] and may thereby affect the outcome of vector competence and virus replication studies. It is therefore important to investigate the presence of ISVs in both cell culture systems and mosquito colonies used for experiments.

In mosquitoes, the primary antiviral response is mediated by small (≤30) non-coding RNAs, that can silence complementary viral RNA [12]. Three main classes of small silencing RNAs can be distinguished: micro (mi)RNAs, small-interfering (si)RNAs, and PIWI-interacting (pi)RNAs (reviewed in [13]). MiRNAs have a length of ~22–23 nts and are produced by the ribonucleases Drosha and Dicer-1 (Dcr1). They are loaded into an Argonaute-1 (Ago1)-containing RNA-induced silencing complex (RISC) to guide recognition of partially complementary target mRNAs, leading to translational repression or degradation [13].

SiRNAs are 21 nts in length and derived from Dcr2 cleavage of double-stranded (ds)RNA of viral or other exogenous origin. SiRNAs are incorporated into an Ago2-RISC complex and guide recognition of fully complementary target RNAs, which are subsequently cleaved by Ago2 and degraded [14,15]. The antiviral activity of the siRNA response has been demonstrated for arboviruses from several genera in various cell culture and mosquito models (reviewed in [13,15]).

The piRNA pathway is known for its function in transposon repression and gene regulation in the germline and has most extensively been studied in *Drosophila*. In *Drosophila*, primary piRNAs are transcribed as precursors from dedicated loci in the genome, termed piRNA clusters. These precursors are processed by the protein Zucchini, loaded into the PIWI-proteins Piwi and Aubergine (Aub), and processed into mature 25–30 nt long piRNAs [16]. Piwi-bound piRNAs target and silence complementary transposons at the transcriptional level [17], whereas Aub-piRNA complexes initiate the ping-pong piRNA amplification cycle [18,19]. In the ping-pong amplification cycle, Aub-bound piRNAs base pair with complementary transposon RNAs. The transposon RNA is then cleaved between position 10–11 complementary to the piRNA sequence. Subsequently, the 3’ cleavage products are transferred to the PIWI-protein Ago3 and processed into mature secondary sense piRNAs of roughly 25–30 nts. These cleavage events produce the typical ping-pong signature, with antisense piRNAs having a 1U bias, sense piRNAs having a 10A bias, and an overlap between sense and antisense piRNAs of 10 nts [18,19].

While *Drosophila* encodes only three PIWI genes, the important arbovirus vectors *Aedes aegypti* and *Culex quinquefasciatus* encode 7 and 6 PIWI genes, respectively [20,21]. This PIWI gene expansion suggests that the piRNA pathway has additional functions in mosquitoes, beyond transposon control and gene regulation in the germline. The recent discovery that some mosquito species produce viral piRNAs (vpiRNAs) during arbovirus infection raises the exciting possibility that this pathway also contributes to host defence against viruses [22,23,24,25,26,27,28,29]. Moreover, as arboviruses replicate in the soma, these observations indicate that, unlike in *Drosophila*, the piRNA pathway is active in the soma of mosquitoes. However, little is known about the mechanisms and functions of the piRNA-pathway in mosquitoes [30]. In *Ae. aegypti*, viral piRNA production is dependent on Piwi5 and Ago3, with a minor contribution of Piwi6, along with the helicase Vasa and the Tudor protein Yb and Veneno [22,23,31]. Antisense 1U-biased viral piRNAs are mainly bound by Piwi5, while most sense 10A-biased viral piRNAs are bound by Ago3 [23].

In *Aedes* spp. mosquitoes or cells, viral piRNAs have been observed during infections of alphaviruses, flaviviruses and bunyaviruses [22,23,25,27,28]. In contrast, in *Cx. quinquefasciatus* mosquitoes arboviral piRNAs have thus far only been described for Rift Valley fever virus, a member of the *Phenuiviridae* family (order *Bunyavirales)* [26], but viral piRNAs have not been observed during alphavirus and flavivirus infection of *Cx. pipiens* [30,32]. It is unclear why different small RNA responses are triggered to viruses from different families, and how the small RNA response to a virus can differ between *Aedes* and *Culex* spp. mosquitoes.

Here, we compared the small RNA responses of cell lines derived from *Cx. tarsalis, Ae. pseudoscutellaris, Ae. albopictus*, and *Ae. aegypti* mosquitoes. *Cx. tarsalis* is a vector mosquito for several arthropod-borne (arbo)viruses including western equine encephalitis virus and West Nile virus (WNV) [33,34]. *Ae. pseudoscutellaris* is an important vector mosquito for parasites that cause human lymphatic filariasis and transmits a limited number of arboviruses including Ross River virus [35]. *Ae. albopictus* is an important vector for many arboviruses including chikungunya virus, dengue and WNV [36] and *Ae. aegypti* acts as the primary vector for human-infecting arboviruses such as Zika, yellow fever, dengue, and chikungunya virus [37]. The small RNA responses to WNV were investigated in *Cx. tarsalis* CT cells, *Ae. pseudoscutellaris* AP-61 cells, *Ae. albopictus* U4.4 and C6/36 cells and *Ae. aegypti* Aag2 cells. Via *de novo* contig assembly of small RNA reads we identified persistent infections with insect-specific viruses (ISVs) from the *Flaviviridae, Phenuiviridae, Birnaviridae, Nodaviridae*, and *Narnaviridae*, which allowed the investigation of small RNA responses across these distinct virus families. In both *Aedes* and *Culex* spp. cells, we observed an siRNA response to all discovered viruses. However, piRNA responses were highly virus-family and cell line dependent. Notably, we observed that the piRNA response to the same virus can differ between *Culex* and *Aedes* mosquito cells, both in regard to the species of small RNAs produced and their relative abundance. These results show that small RNA responses are highly host species and virus family dependent.

## 2. Materials and Methods

### 2.1. Cell Culture and Virus Infections

*Ae. albopictus* C6/36 (ATCC CRL-1660) and U4.4 cells [38] were cultured at 28 °C in Leibovitz-L15 medium (Gibco, Carlsbad, CA, USA) supplemented with 10% heat-inactivated fetal bovine serum (FBS; Gibco), 2% tryptose phosphate broth (Gibco) and 1% nonessential amino acids (Gibco). *Ae. aegypti* Aag2 cells [39], *Ae. pseudoscutellaris* AP-61 cells [40], and *Cx. tarsalis* CT cells (provided by Dr. Aaron Brault, Centers for Disease Control and Prevention, Fort Collins, CO, USA) [41] were cultured at 28 °C in Schneider’s *Drosophila* medium (Gibco) supplemented with 10% FBS. The infectious clone used to generate WNV lineage 2, originally from south eastern Europe, was described before [42]. Briefly, pre-seeded 6-well plates of Vero cells were transfected with ~1 µg of WNV plasmid DNA using Lipofectamine 2000 (Invitrogen, Carlsbad, CA, USA) to generate the P0 virus stock. Subsequent passages were performed on C6/36 cells and the P2 stock was used for experiments. End-point dilution assays (EPDA) on Vero cells were used to determine the virus titer (tissue culture infectious dose 50% (TCID_50_)/mL), as previously described [42].

### 2.2. Eradication of Cell Fusing agent Virus from Aag2 Cells

Aag2 cells were cleared of persistent cell fusing agent virus (CFAV) infection by prolonged treatment with dsRNA targeting CFAV. DsRNA was prepared by *in vitro* transcription using the RiboMAX T7 system (Promega, Madison, WI, USA) on PCR templates with flanking T7 promoters made using Phusion DNA polymerase (New England Biolabs, Ipswich, MA, USA) (primer sequences are provided in Table S1). CFAV PCR products were generated from Aag2 cells total RNA followed by reverse transcription with cDNA TaqMan reverse transcription reagents (Thermo Fisher, Waltham, MA, USA) and pGL3 plasmid DNA was used as a template for the luciferase control. Aag2 cells were seeded at a density of 5 × 10^5^ cells per well in 24-well plates and transfected the following day with 150 ng dsRNA targeting either luciferase (GL3; ~400 bp) as a control, CFAV NS1 (dsNS1; ~300 bp), CFAV NS5 (dsNS5; ~300 bp), or a combination of both dsNS1 and dsNS5 using X-tremeGENE HP (Roche, Basel, Switzerland) according to the manufacturer’s recommendations. Two to three days after transfection the cells were washed with phosphate buffered saline (PBS), resuspended, and transferred to a new 24-well plate. The following day the cells were transfected as described above. After six sequential dsRNA transfections, cells transfected with CFAV dsRNA were washed with PBS, resuspended, and transferred to 96-well plates at a density of a single cell per well and 100 ng of dsRNA was added to each well without any transfection reagent. Four weeks after seeding, confluent clones were expanded for further analysis. Total RNA was isolated using RNA-Solv reagent (Omega Biotek, Norcross, GA, USA), and CFAV RNA levels were analysed by reverse-transcription (RT)-qPCR using cDNA TaqMan reverse transcription reagents and GoTaq qPCR master mix (Promega) according to the manufacturer’s recommendations. Presence of Culex Y virus and Phasi Charoen-like virus in the cell clones was analysed by end-point PCR. CFAV-free cell clone Aag2-C3 was used for WNV infection and small RNA analyses. 

### 2.3. RNA Isolation and Deep Sequencing of Small RNAs

Monolayers of mosquito cells were either left untreated or infected with WNV at a multiplicity of infection (MOI) of 1 in 6-well plates. RNA from untreated cells and WNV-infected cells was isolated at four days post infection using TRIzol reagent (Invitrogen) following the manufacturer’s protocol. Small RNA deep sequencing libraries for WNV-infected C6/36, U4.4 and CT cells were prepared as described previously [42]. Briefly, 15 µg total RNA was size-separated by electrophoresis on 15% polyacrylamide, 7 M Urea, 0.5X Tris-boric acid EDTA (TBE) gels. The 19–30 nt RNAs were excised from gel and eluted in 800 µL 0.3 M sodium acetate, precipitated with 80% ethanol and dissolved in 11 µL H_2_O. Small-RNA libraries were prepared using the TruSeq small-RNA kit (15016914; Illumina, San Diego, CA, USA) and sequenced on an Illumina HiSeq 2500 sequencer (Baseclear, Leiden, The Netherlands). Single-end FASTQ reads were generated with the Illumina Casava pipeline version 1.8.3. Initial quality assessments were performed using in-house filtering protocols of Baseclear and the FASTQC quality control version 0.10.0. Small RNA libraries from mock-infected cells and the WNV-infected AP-61 and Aag2 cells were generated from ~1 µg total RNA on a BGISEQ-500 sequencing platform (BGI Group, Shenzhen, Guangdong, China). Single-end FASTQ reads were generated with an in-house filtering protocol of BGI. Small RNA sequencing libraries have been uploaded to the NCBI sequence read archive (SRA) under BioProject PRJNA525601.

### 2.4. Small RNA Analysis

Small RNA sequencing libraries were analysed on the Galaxy webserver [43]. Adapters were trimmed using the Clip tool version 10.1. Reads were mapped with Bowtie2 [44] version 2.3.4.2 allowing 1 mismatch with a seed length of 28. Reads were mapped to the viral genomes of WNV, Culex Y virus (A-fragment JQ659254.1; B-fragment JQ659255.1), Flock House virus (RNA-1 JF461541.1; RNA-2 JF461542.1), Phasi-Charoen like virus (L-segment KU936057.1; M-segment KU936056.1; S-segment KU936055.1), Narnavirus (KP642120.1), *Aedes* Densovirus (MH550148.1), and Calbertado virus (KX669689.1). To identify reads derived from the genome of Aag2 cells, reads were mapped to the *Ae. aegypti* Aag2 cell line contig assembly [45]. Similarly, C6/36 cell genome derived sequences were identified by mapping to the C6/36 contig assembly [46]. Genome distributions of siRNAs and piRNAs were produced by filtering reads with a length of 21 or 25–30 nts respectively and mapping the genome positions of the 5’ end of each read. Sequence signatures were generated using Weblogo3 from mapped sense and reverse-complemented antisense reads that were 3’ trimmed to 20 nts. Sequence overlaps of piRNAs were determined using the small RNA signatures tool [47] version 3.1.0 on the mississippi.snv.jussieu.fr Galaxy server using 25–30 nt reads as input.

### 2.5. De Novo Contig Assembly from Small RNA Reads

*De novo* contig assembly of small RNA reads was performed with the Metavisitor Oases/Velvet pipeline with k-mers ranging from 15–29 on the mississippi.snv.jussieu.fr Galaxy server on clipped reads, as described in [48,49]. Retrieved contigs were filtered for a minimum length of 200 nts. Contigs were blasted with the BLASTx tool in the NCBI plus toolkit to all sequences in the NCBI database that match the criteria “txid10239[orgn] NOT txid131567[orgn] NOT phage[title]”, which retrieves all non-phage virus sequences. Contigs with a viral blast hit were assembled using the Contigassembler tool in Vector NTI 11.0 (Invitrogen), and retrieved contigs were blasted against the NCBI non-redundant nucleotide database with BLASTn to identify the viral origin. The complete genome sequence or CxNV1 has been uploaded to GenBank under accession number MK628543.

## 3. Results

### 3.1. Small RNA Profiles in Uninfected and WNV-Infected Aedes and Culex spp. Mosquito Cells

**Figure 1:**
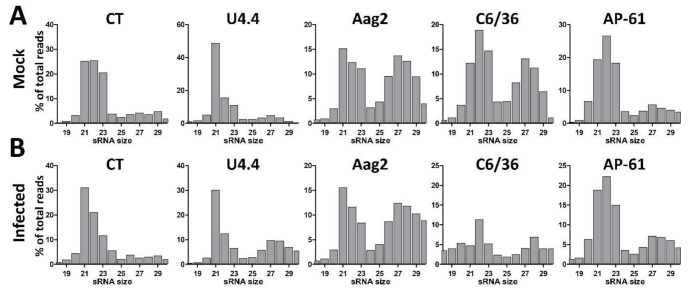
Small RNA profiles of Mock-infected and WNV-infected CT, U4.4, Aag2, C6/36 and AP-61 mosquito cells. Small RNA sequencing libraries of (**A**) Mock-infected and (**B**) West Nile virus infected cells. Shown is the small RNA size distribution of 18–30 nt reads, normalized to the total number of reads in each library.

To investigate the small RNA responses of *Aedes* and *Culex* cells to flavivirus and insect-specific virus infection, we deep sequenced the small RNAs of uninfected and WNV-infected *Cx. tarsalis* CT cells, *Ae. aegypti* Aag2 cells, *Ae. albopictus* U4.4 and C6/36 cells, and *Ae. pseudoscutellaris* AP-61 cells. Libraries were prepared from 18–30 nt small RNAs and size-distributions were generated and normalized to the total number of reads per library (Figure 1). In the uninfected cells a peak of 21 nt small RNAs, likely corresponding to the population of siRNAs, was clearly observed in U4.4 and Aag2 cells (Figure 1A). In CT, C6/36, and AP-61 cells, 21 nt small RNAs were also observed, but their proportion was lower relative to the population of 22–23 nt small RNA, which likely corresponds to miRNAs (Figure 1A). A population of 25–30 nt small RNAs, which is in the size range of piRNAs, was clearly observed in Aag2 and C6/36 cells, and to a lesser extent in the other cell lines (Figure 1A). No significant changes in small RNA profiles were observed upon WNV-infection, except for CT cells where the 21 nt siRNA peak became more pronounced (Figure 1B). In WNV-infected C6/36 cells, the percentage of 19–30 nt reads was decreased, likely due to an overrepresentation of a 15 nt sequence in the sequence library (18% of total reads) with 100% identity to three putative *Aedes* RNAs (XR_002500242.1; XR_002500243.1; XM_021851720.1).

### 3.2. Discovery of Insect-Specific Viruses by De Novo Assembly of Small RNA Reads

We investigated the presence of ISVs in the uninfected CT, U4.4, Aag2, C6/36 and AP-61 cells by *de novo* assembly of small RNA reads, using a pipeline previously described by Carissimo *et al.* [48,49]. Assembled contigs were filtered for a minimal size of 200 nts and compared to viral sequences in the NCBI database using BLASTx. Contigs with a viral blast hit, an indication of a potential virus-derived sequence, were re-assembled to produce longer sequences, resulting in 32, 46, 153, 72 and 19 assemblies for CT, U4.4, Aag2, C6/36, and AP-61 cells, respectively (Table 1). In CT cells, assemblies were found corresponding to the genomes of Flock House virus (FHV), Calbertado virus (CLBOV), Phasi Charoen-like virus (PCLV) and a narnavirus (Table 2). The narnavirus has 97% sequence identity to an unnamed narnavirus detected by NGS in *Culex pipiens* [50] and will be referred to as Culex narnavirus 1 (CxNV1). The full CxNV1 genome sequence is available in GenBank (MK628543). CLBOV, PCLV, and CxNV1 contigs covered >65% of the viral genome, suggesting that these viruses replicate in CT cells. However, for FHV a low coverage of 12% and 24% was reached for RNA-1 and RNA-2, respectively.

In U4.4 cells, contigs were retrieved corresponding to CYV with a coverage of 100%, indicating that CYV is replicating in these cells. In Aag2 cells, contigs of PCLV and *Aedes* densovirus (AeDNV) were recovered (Table 2). For PCLV, contigs were only retrieved corresponding to the M- and S-segments but not the L-segment of PCLV and the coverage of AeDNV was only 23%. Aag2 cells have previously been demonstrated to be persistently infected with cell fusing-agent virus (CFAV) [2,51] and CYV [52]. However, our Aag2 cell line has been repeatedly transfected with CFAV-complementary dsRNA corresponding to ~320 bp of the NS1 protein, NS5 protein or both, to clear CFAV from these cells (Figure S1). Surprisingly, CYV, which persistently infected the parental Aag2 cell line, was also lost from the cells during this procedure, and the level of PCLV infection was decreased as compared to the parental cell line (Figure S1). Possibly, the continuous treatment with dsRNA resulted in cellular stress or activation of antiviral mechanisms which interfered with CYV and PCLV replication. In accordance, <10 reads mapped to the CFAV and CYV genomes, confirming that our Aag2 cells are CFAV- and CYV-free.

In addition to exogenous viruses, endogenous viral elements (EVE), also referred to as non-retroviral integrated RNA virus sequences (NIRVS), can be a source of viral small RNA sequences [15,45,53,54,55]. The relatively low coverage for FHV in CT cells, and PCLV and AeDNV in Aag2 cells therefore indicates either that these viruses are replicating at a very low level, or that these contigs were assembled from EVE-derived small RNAs. Alternatively, viruses may produce defective-interfering (DI) RNAs, that are partial viral genomes containing rearrangements and/or deletions, which may result in a skewed small RNA population towards those regions that are more prevalent in DIs [56,57,58].

### 3.3. Small RNA Profiles of Insect-Specific Viruses in Culex Tarsalis cells Reveal the Production of Viral siRNAs and piRNAs

**Figure 2:**
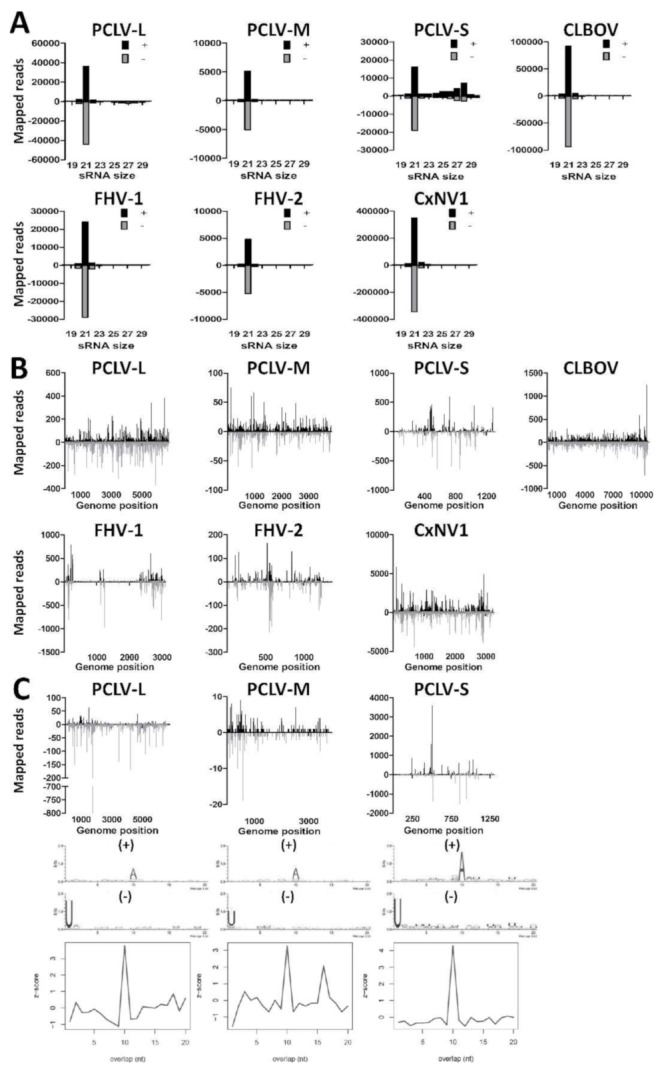
Small RNA responses to insect-specific viruses in CT cells. (**A**) Size distribution of 18–30 nt small RNAs that map to the genomes of Phasi Charoen-like virus (PCLV), Calbertado virus (CLBOV), Flock House virus (FHV), or Culex narnavirus 1 (CxNV1). Shown is the number of reads mapping to the sense (+; black) or antisense (−; gray) genome. (**B**) Genome distributions of 21 nt small RNAs that map to the genomes of PCLV, CLBOV, FHV, or CxNV1. (**C**) Genome distributions, sequence logos and the probability of overlap length of 25–30 nt small RNAs that map to the genome of PCLV.

As contigs were found for PCLV, CLBOV, FHV, and CxNV1 by *de novo* assembly of small RNAs from CT cells (Table 2), we investigated the small RNA profiles to these viruses. We first mapped the small RNAs of the CT cells to the ISV genomes and generated small RNA size distributions for the (+) and (−) strands (Figure 2A). Interestingly, large numbers of 21 nt viral siRNAs aligned to the (+) and (−) strands of PCLV, CLBOV, FHV, and CxNV1. Next, we mapped the 21 nt viral siRNAs along the viral genomes for both the (+) and (−) strands (Figure 2B). For PCLV, CLBOV and CxNV1 the viral siRNAs were distributed all along the viral genome. For FHV the viral siRNAs were found to map predominantly to specific hotspots on the viral genome, similar to previous reports of FHV-infected *Drosophila* cells, suggesting that these are derived from FHV DIs [57,59,60]. Viral siRNAs that mapped to the S-segment of PCLV were predominantly found in several hotspots. The distribution of small RNAs along the viral genomes in combination with a high coverage after *de novo* assembly indicates that PCLV, CLBOV, FHV, and CxNV1 replicate in CT cells.

Curiously, a shoulder of 25–30 nt viral piRNAs was found to map to both the (+) and (−) strand of the PCLV S-segment (Figure 2A), suggesting that CT cells are capable of producing pi-like RNAs, despite the absence of viral piRNAs in previous reports from *Culex* mosquitoes [30,32]. Similarly, pi-like RNAs were also found to map to the PCLV M- and L-segments, although they were much less abundant and therefore not observed as a clear piRNA shoulder. Mapping of the 25–30 nt small RNAs along the PCLV L-, M-, and S-segments revealed that these were derived from hotspots on the (+) and (−) strands (Figure 2C). These 25–30 nt putative-viral piRNAs displayed 10A (+) and 1U (−) biases in combination with a 10 nt overlap between the small RNAs derived from the (+) and (−) strands, indicative of ping-pong amplified viral piRNA production to this particular ISV. However, no evidence of viral piRNAs was found for CLBOV, FHV, nor CxNV1 in CT cells, indicating that the formation of viral piRNAs is highly virus-specific. These results show that CT cells support replication of ISVs from distinct viral families despite the potent RNAi response in these cells, and are capable of producing viral piRNAs.

### 3.4. U4.4 Cells are Persistently Infected with Culex Y Virus and Demonstrate a Potent siRNA Response

**Figure 3:**
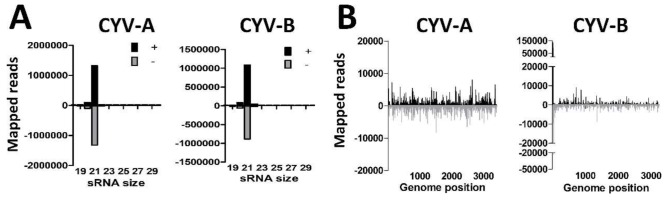
Small RNA responses against Culex Y virus in U4.4 cells. (**A**) Size distribution of 18–30 nt small RNAs that map to the genome of Culex Y virus (CYV). Shown is the number of reads mapping to the sense (+; black) or antisense (−; gray) genome. (**B**) Genome distributions of 21 nt small RNAs that map to the genome of CYV.

*De novo* contig assembly of small RNAs implicated the presence of CYV in U4.4 cells. We therefore mapped the small RNAs from U4.4 cells to the (+) and (−) strands of the CYV genome, and analysed the small RNA size-distributions (Figure 3A). A high number of reads mapped to both the A- and B- segments of the CYV genome in agreement with the results of the *de novo* assembly (Table 2). Strong 21 nt viral siRNA peaks were observed that mapped to both the (+) and (−) strands of the A- and B-segments of CYV (Figure 3A), indicating that U4.4 cells generate an siRNA response to this virus. Mapping of the 21 nt viral siRNAs along the genome segments of CYV indicated that viral siRNAs are distributed across the A- and B-segments of CYV (Figure 3B). These results implicate that U4.4 cells are indeed persistently infected with CYV.

### 3.5. Aag2 Cells Produce siRNAs and piRNAs to Insect-Specific Virus Infection

**Figure 4:**
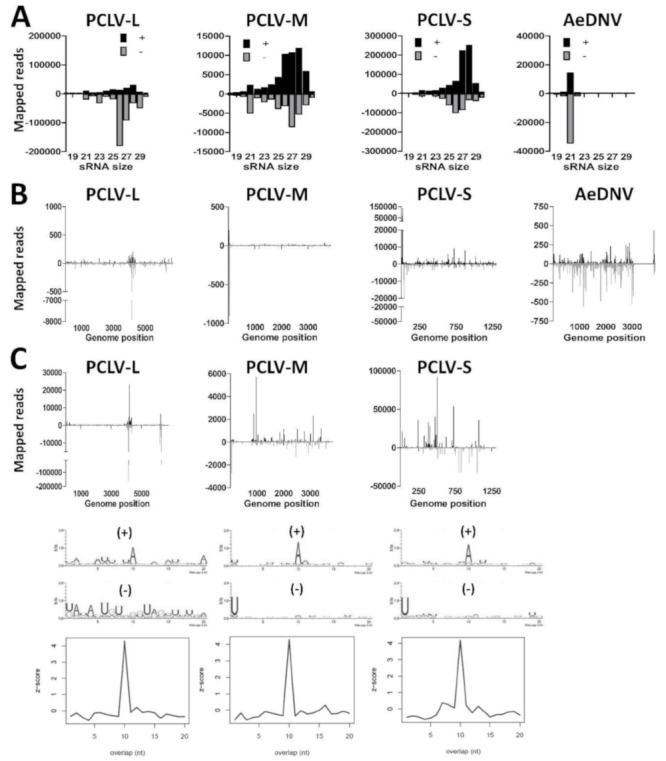
Small RNA responses against insect-specific viruses in Aag2 cells. (**A**) Size distribution of 18–30 nt small RNAs that map to the genomes of Phasi Charoen-like virus (PCLV) or *Aedes* densovirus (AeDNV). Shown is the number of reads mapping to the sense (+; black) or antisense (−; gray) genome. (**B**) Genome distributions of 21 nt small RNAs that map to the genomes of PCLV or AeDNV. (**C**) Genome distributions, sequence logos and the probability of overlap length of 25–30 nt small RNAs that map to the genome of PCLV.

We investigated the small RNA responses to PCLV and AeDNV in Aag2 cells by mapping the small RNAs to the respective viral genomes (Figure 4A). For PCLV, primarily 25–30 nt small RNAs mapped to the genome, suggesting the production of PCLV-derived piRNAs, while few 21 nt PCLV siRNAs were observed. Curiously, for AeDNV, only siRNAs, but no piRNAs were observed. We then mapped the 21 nt viral siRNAs along the genomes of PCLV and AeDNV (Figure 4B). For PCLV, siRNAs mapped primarily to specific hotspots on the L-, M-, and S-segment, with a lower number of reads distributed across the genome. For AeDNV the siRNAs mapped evenly along the viral genome, except for a gap near the 3’ end, corresponding to a region in the AeDNV capsid coding region. To investigate whether these PCLV and AeDNV small RNA reads may be derived from EVEs, we depleted our small RNA library for RNAs that mapped to the Aag2 genome and re-mapped the remaining reads to the PCLV and AeDNV genome. No clear changes were observed in the PCLV small RNA size profiles, indicating that the PCLV small RNAs are not derived from EVEs (Figure S2). However, for AeDNV all small RNA reads mapped to the Aag2 genome, implicating that the AeDNV small RNA reads are possibly derived from one or more EVEs that cover nearly the entire AeDNV genome. We can therefore not conclude that AeDNV is replicating in our Aag2 cells.

We then mapped the 25–30 nt putative PCLV-derived piRNAs to the L-, M-, and S-segments (Figure 4C). Again, reads mapped predominantly to two hotspots on the L-segment, at similar locations as the siRNA hotspots. For the M- and S-segments reads mapped to hotspots along the viral genome. Analysis of sequence bias and overlap between sense and antisense reads indicated a typical ping-pong piRNA signature for piRNAs that mapped to the PCLV L-, M-, and S-segment, suggesting that these small RNAs are indeed PIWI-interacting and amplified by the ping-pong cycle. These results show that Aag2 cells are persistently infected with PCLV and are capable of producing viral siRNAs and *bona fide* viral piRNAs.

### 3.6. Differential Small RNA Responses to WNV-Infection in Culex and Aedes Mosquito Cells

**Figure 5:**
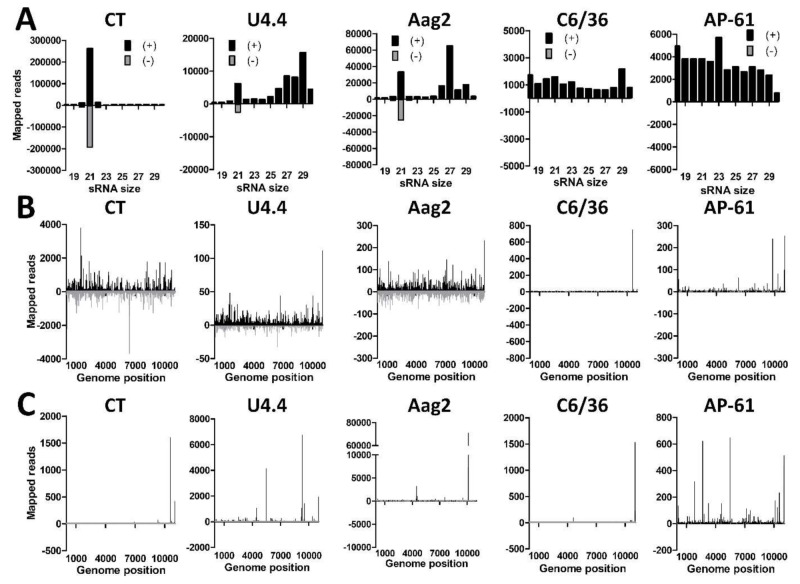
Small RNA responses to West Nile virus in *Culex* and *Aedes* spp. cells. (**A**) Small RNA reads of CT, U4.4, Aag2, C6/36, and AP-61 cells were mapped to the West Nile virus (WNV) genome. Shown is the small RNA size distribution of sense (+; black) and antisense (−; gray) mapping reads of 18–30 nts for each cell line normalized to the total number of reads in each library. (**B**) Distribution of mapped sense and antisense 21 nt small RNAs along the WNV genome in each cell line. (**C**) Genome distribution of sense and antisense 25–30 nt small RNAs that map to the WNV genome in each cell line.

To assess the small RNA responses to WNV in different types of mosquito cells, we infected CT, U4.4, Aag2, C6/36, and AP-61 cells with WNV lineage 2 at an MOI of 1. At 4 dpi the WNV titer was determined by end-point dilution assay and RNA was isolated and subjected to small RNA deep sequencing. WNV titers of 7.1 × 10^7^, 9.6 × 10^7^, 8.7 × 10^9^, 3.2 × 10^8^ and 1.1 × 10^8^ TCID_50_/mL were determined from the supernatant of CT, U4.4, Aag2, C6/36, and AP-61 cells, respectively, indicating that WNV replicated to high levels in all cell lines. We then mapped the small RNA reads from the WNV-infected cell lines to the sense (+) and antisense (−) strand of the WNV genome. Small RNA size-distributions were generated and normalized to the total number of reads (Figure 5A). In CT cells, a strong 21 nt peak was observed on both the (+) and (−) strand, indicative of a potent siRNA response to WNV. In U4.4 and Aag2 cells a similar 21 nt siRNA peak was observed on the (+) and (−) strand, albeit less evident as in CT cells. However, no 21 nt siRNA peak was observed in the Dcr2 deficient C6/36 cells [61]. Of note, siRNAs were also absent in AP-61 cells, suggesting that these cells may have a similar deficiency in Dcr2 cleavage. Mapping the 21 nt viral siRNAs along the WNV genome demonstrated that they target the entire WNV genome in CT, U4.4, and Aag2 cells, although hot and cold spots could be distinguished (Figure 5B). The few 21 nt reads in C6/36 and AP-61 cells mapped mostly to the positive strand of the WNV genome at few specific locations near the 3’ end. This again suggests that both C6/36 and AP-61 cells have a deficiency in their RNAi machinery and are unable to produce a clear 21 nt viral siRNA response.

A clear shoulder of 25–30 nt small RNAs was observed in U4.4 and Aag2 cells that mapped to the WNV (+) strand but not to the (−) strand, indicative of viral piRNAs (Figure 5A). This population of putative piRNAs was much smaller in CT, C6/36 and AP-61 cells. Mapping of the 25–30 nt putative viral piRNAs from all the mosquito cell lines to the WNV genome indicated that they target specific hotspots in the viral genome (Figure 5C), similarly as demonstrated for other flaviviruses [22,25]. To further investigate whether the 25–30 nt small RNAs in CT and U4.4 cells could represent *bona fide* viral piRNAs, sequence logos and overlap signatures were generated of these putative viral piRNA populations. No 1U or 10A bias nor 10-nt overlap was observed for the 25–30 nt small RNAs (Figure S3) and it is thus unclear whether or not they are interacting with PIWI proteins.

## 4. Discussion

Culicine mosquitoes have an expanded repertoire of PIWI genes and it is currently unknown to which extent the piRNA responses of *Drosophila* and mosquitoes or even different mosquito species have shared functions and characteristics [20,21]. For example, while *Aedes* spp. mosquitoes and cells are capable of producing viral piRNAs in response to alphavirus and flavivirus infection [22,23,25], no viral piRNAs have so far been observed to these viruses in *Culex* spp. mosquitoes [30,32]. In the present study, we observed WNV pi-like RNAs in *Ae. albopictus* U4.4 and *Ae. aegypti* Aag2 cells. This is in line with previous studies with DENV and ZIKV in *Ae. aegypti* Aag2 cells, where pi-like RNAs were equally (+) strand biased and lacked a ping-pong signature [22,25]. DENV pi-like RNAs in Aag2 cells are dependent on Piwi5 and Ago3, implicating that these populations of 25-30 nt flavivirus pi-like RNAs are produced by the piRNA machinery and can be considered as true piRNAs [22]. Strikingly, we observed no significant WNV, CLBOV, FHV, or CxNV1 pi-like RNAs in CT cells, suggesting that *Culex* mosquito cells do not produce viral piRNAs in response to acute WNV-infection and infection with other persistently infecting positive strand RNA viruses. In contrast, PCLV-derived viral piRNAs were readily detected in CT cells, indicating that *Cx. tarsalis* cells are intrinsically capable of producing viral piRNAs. In U4.4 cells, WNV-derived pi-like RNAs were produced only against the (+) strand and did not have a ping-pong signature, while no pi-like RNAs were produced against CYV. In Aag2 cells, PCLV-derived piRNAs had a clear ping-pong signature and WNV-derived piRNAs lacked a ping-pong signature. Moreover, while PCLV-derived viral piRNAs were observed in both CT and Aag2 cells, the ratio between viral piRNAs and siRNAs was substantially higher in Aag2 cells. A higher piRNA:siRNA ratio in *Aedes* compared to *Culex* mosquitoes was also observed in a previous study with Rift Valley fever virus, which like PCLV belongs to the family *Phenuiviridae* [26].

The differential small RNA responses for different combinations of cell lines and virus infections implicate two important findings. Firstly, these observations suggest differences in the small RNA responses of *Aedes* spp. versus *Culex* spp. mosquitoes and derived cell lines. These differences may be caused by differential availability of viral genomic RNA to the small RNA machineries in *Aedes* compared to *Culex* cells, which could be due to alternate virus replication characteristics or compartmentalization of replication complexes in *Aedes* versus *Culex* cells. Furthermore, as details on the functions of *Aedes* and especially *Culex* PIWI proteins remain largely unknown, differences in the piRNA machinery itself may underlie variation in piRNA production between *Culex* and *Aedes* mosquitoes. Moreover, for most cell lines it is unknown which cell type or organ they represent due to the method used for cell line generation. For example, the C6/36 cell line was generated by twice selecting *Ae. albopictus* larvae-derived Singh cells [38] for the highest virus yield of Sindbis virus (clone 6 and clone 36, respectively) [62]. U4.4 cells were originally derived from freshly hatched *Ae. albopictus* larvae [38] and AP-61 cells were generated from freshly hatched *Ae. pseudoscutellaris* larvae [63]. As these cell lines originally contained cells from various organs, possible differences in small RNA responses across mosquito cell lines may thus represent variation in the small RNA machineries of cells from diverse mosquito tissues.

Secondly, the different small RNA responses to different viruses in a single cell line suggests that the production of viral piRNAs in a particular mosquito species is largely virus family dependent. Such differences in viral piRNA production in a single cell model may be caused by the viral coding strategy, as the genome organization of the investigated viruses is quite different (WNV/FHV/CLBOV/CxNV1, (+) ssRNA; PCLV, (−) ssRNA; CYV, dsRNA). Thus, it is possible that differences in small RNA profiles can also be explained by differences in the availability of the viral RNAs as substrate for the piRNA machineries due to the viral coding and replication strategies.

In contrast to the variation in viral piRNA responses, abundant siRNAs were detected for all viruses in U4.4, Aag2 and CT cells. For WNV, CYV, PCLV, and CxNV1, siRNAs mapped all along the viral genome in hot and cold-spots. The hot-spot siRNA and piRNA mapping patterns implicate the importance of RNA-accessibility for the production of small RNAs for most viruses in mosquito cells [22,23,25,26,27,29,32,42,64]. Hot-spots are potentially more prone to targeting by small RNA machineries due to the formation of preferentially targeted secondary RNA structures. In contrast, cold-spots may be targeted less due to their association with viral or host proteins, or the formation of tight RNA folding that makes these areas less accessible for nuclease cleavage. Alternatively, the production of small RNAs from DI RNAs and/or EVEs can skew the small RNA populations to certain regions of the viral genome, which may also be observed as cold- and hotspots [15,45,53,54,55,56,57,58].

Despite the presence of abundant siRNAs to CYV, FHV, PCLV, CLBOV, and CxNV1, the assembly of nearly full-length genomes of these viruses suggests that they are still able to replicate persistently in mosquito cell lines. It remains enigmatic how viruses can maintain a persistent infection in cells that contain such abundant virus-specific siRNAs. Possibly, these persistent viruses hide their replication complexes in virus-induced membranes [65,66] and/or they replicate at a low level, due to the continuing degradation of viral RNA by the host defence systems, resulting in the production of small RNAs. Of note, not all siRNAs may actually be functionally silencing, as it has previously been described for some viruses that hot-spot derived siRNAs are less efficient in silencing [59,67].

We did not observe an siRNA response to acute WNV infection of either AP-61 nor C6/36 cells, suggesting that AP-61 cells, like C6/36 cells [61], are siRNA-deficient. The total small RNA libraries of both C6/36 cells and AP-61 cells did, however, contain small RNAs with a length of 21 nts. Possibly, these may represent miRNAs which can have a length of 21 nts, although the majority of miRNAs is expected to be ~22–23 nts [68]. Alternatively, these 21 nt reads may be derived from host RNAs, likely through Dcr2 independent cleavage, and may partially represent residual RNA degradation products. Nonetheless, the absence of siRNAs to acute WNV-infection in both C6/36 and AP-61 cells clearly demonstrates the inability of these cells to generate an siRNA response to virus infection.

Notably, despite the presence of ISVs in the siRNA-competent CT, U4.4 and Aag2 cells, we could not detect replicating ISVs in C6/36 cells, similar to recent studies that did not detect ISVs in C6/36 cells [2,52,69]. Curiously, also no ISV infections were detected in AP-61 cells. The correlation between siRNA-deficiency and the absence of persistent viruses is somewhat counterintuitive, and is not mechanistically understood. It has recently been postulated that the persistent infection of mosquito cells may require an active siRNA response [58,70]. The rationale behind this is that the siRNA response suppresses viral replication to a level that is tolerable to the host cell, thereby sustaining the persistent infection with no or minimal cytopathic effects. Thus, it is possible that C6/36 and AP-61 cells mainly support acute virus infections and that persistence is more difficult to establish. However, persistent infections of arboviruses have been reported in C6/36 [71,72,73] and AP-61 cells [74], thus, an siRNA response may not be strictly required for the establishment of persistent infections. Alternatively, the apparent lack of ISV infections in the siRNA-deficient C6/36 and AP-61 cells may be inherent to our small RNA sequencing approach, as viruses to which only siRNAs are produced may no longer be detected in an siRNA-deficient cell line. Nonetheless, the absence of ISVs in other studies that use different methods for virus detection [2,52,69] suggests that siRNA-deficient mosquito cell lines are not commonly infected with ISVs.

We discovered CxNV1 in our CT cells, which has the closest homology to a previously discovered narnavirus by NGS in field-caught *Cx. pipiens* mosquitoes [50]. This finding is surprising, as viruses from the *Narnaviridae* family have so far only been shown to infect yeast and oomycetes [75]. As mosquitoes can carry a variety of fungi and bacteria, possibly CxNV1 replicates in mosquito-associated yeast. However, there were no signs of fungal or bacterial contamination in our CT cells. Furthermore, the assembly of a full-length genome of CxNV1, and the presence of typical 21-nt viral siRNAs, suggests that CxNV1 replicates in CT cells. Interestingly, while narnaviruses encode a single protein that serves as the viral RNA-dependent RNA polymerase [75], CxNV1 encodes an additional ORF on the minus strand of no known homology. A similar ambisense coding strategy of narna-like viruses was recently described in other insect-associated viruses by NGS [76]. It is therefore possible that CxNV1 is a member of a new group of insect-specific, narna-like viruses which have incorporated a second ORF to facilitate infection of insects.

In conclusion, we have demonstrated that CT, U4.4, and Aag2 cells are capable of producing viral siRNAs and piRNAs. This establishes CT cells as the first model cell line to analyse *Culex* piRNAs. Moreover, the divergent production of viral piRNAs in *Aedes* versus *Culex* mosquito cell lines to the same virus, suggests functional variation in the piRNA machineries of *Culex* and *Aedes* mosquitoes. Furthermore, differences in small RNA profiles for viruses from distinct viral families implicate an important role of the viral coding and replication strategy in the targeting by small RNA machineries. These results contribute to our understanding of the mosquito small RNA machineries and their implications for arbovirus and ISV infections.

## Figures and Tables

**Table 1:** *De novo* contig assembly of small RNA reads. Small RNA reads from Mock-infected CT, U4.4, Aag2, C6/36, and AP-61 cells were used as input for *de novo* assembly of small RNAs. Shown are the number of small RNAs per library, the retrieved number of contigs from the *de novo* assembly pipeline before and after filtering for length and viral origin, and the final number of assemblies per cell line.

Cell Line	Small RNAs	Contigs	Contigs >200 nts	Virus Blastx Hits	Assemblies
CT	37,450,526	2,126	1,201	198	32
U4.4	37,470,471	3,423	1,655	296	46
Aag2	37,075,343	5,969	3,357	736	153
C6/36	37,769,699	4,393	2,267	322	72
AP-61	29,104,543	1,435	615	83	19

**Table 2:** Insect-specific viruses discovered by *de novo* contig assembly of small RNA reads. Listed are the viruses for which a contig blast-hit was retrieved. Shown is the genome size of the subject, the nucleotide coverage of query contigs, the coverage expressed as percentage of query nucleotide coverage length compared to the length of the subject, the number of mismatching nucleotides between the query and the subject, and the percentage nucleotide identity between the query and the subject sequence.

Virus	Virus Family	Genome	Segment	Genome Size (nts)	Cell Line	Coverage (nts)	Coverage (%)	Mismatches (n)	Identity (%)
CYV	*Birnaviridae*	dsRNA	A	3,429	U4.4	3,429	100	38	99
			B	3,254	U4.4	3,254	100	30	99
PCLV	*Phenuiviridae*	-ssRNA	L	6,769	CT	6,671	99	114	98
					Aag2	0	0	N/A	N/A
			M	3,849	CT	2,628	68	54	98
					Aag2	3,213	83	2	100
			S	1,362	CT	1,362	100	62	95
					Aag2	345	25	0	100
CLBOV	*Flaviviridae*	+ssRNA	N/A	10,735	CT	7,674	71	21	100
FHV	*Nodaviridae*	+ssRNA	1	3,107	CT	359	12	17	95
			2	1,365	CT	333	24	23	93
CxNV1	*Narna-like*	+ssRNA^*^	N/A	3,105	CT	3,105	100	81	97
AeDNV	*Parvoviridae*	ssDNA	N/A	3,937	Aag2	913	23	41	96

* The discovered narnavirus-like virus appears to have an ambisense coding strategy, but is referred to as a (+)-strand RNA virus due to its phylogenetic connection with the *Narnaviridae* family.

## Supplementary Materials

The following are available online at https://www.mdpi.com/1999-4915/11/3/271/s1, Figure S1: Clearance of cell fusing-agent virus (CFAV) and Culex Y virus (CYV) from Aag2 cells, Figure S2: Small RNA reads from uninfected Aag2 cells that map to the genomes of PCLV and AeDNV after depletion of host-derived small RNAs, Figure S3: CT, U4.4, Aag2, C6/36 and AP-61 mosquito cells do not produce WNV piRNAs, Table S1: Sequences of primers used.
