# Supplementary material for: Mosquito Small RNA Responses to West Nile and Insect-Specific Virus Infections in Aedes and Culex Mosquito Cells
Source: Viruses. 2019 Mar 18;11(3):271. doi: 10.3390/v11030271 (PMC6466260; doi:10.3390/v11030271)
Supplement: Supplementary file 1 [file viruses-11-00271-s001.zip › Table_S1.docx]

Table S1. Sequences of primers used.

| Name | Sequence (5’→3’) |
| --- | --- |
| T7-GL3 87 fw | TAATACGACTCACTATAGGGAGATATGAAGAGATACGCCCTGGTT |
| T7-GL3 529 rev | TAATACGACTCACTATAGGGAGATAAAACCGGGAGGTAGATGAGA |
| T7-CFAV NS1 603 Fw | TAATACGACTCACTATAGGGAGAGGCGTTGTCGTGAAGAATGG |
| T7-CFAV NS1 917 Rv | TAATACGACTCACTATAGGGAGAACCTTGACCTTTGTGCCAGG |
| T7-CFAV NS5 2110 Fw | TAATACGACTCACTATAGGGAGAGCCTGAAAGACGGCAGAATC |
| T7-CFAV NS5 2434 Rv | TAATACGACTCACTATAGGGAGAGAATCCACGGGTTGTTGTGC |
| Aaeg RpL5-1 Fw | TCACCTGCCAGATTGCGTACGCCCG |
| Aaeg RpL5-1 Rv | GCTTCTGCAGGATGCGGCGGGCAA |
| CFAV NS1 47 Fw | GCAGCGGCGCTTTTGTGTGG |
| CFAV NS1 201 Rv | GCACTGCAAGGCATCCTCAC |
| CxYV VP1 159 Fw | GCACTACTTGAGCACTCGGT |
| CxYV VP1 323 Rv | GGTAGTATTTGTCCGGCCCC |
| PCLV RdRp 46 Fw | ATTCAGGGGGATGTTGTCAC |
| PCLV RdRp 197 Rv | CCGACAGTAGAAACCACGTC |
