# Supplementary figures and images for: Mosquito Small RNA Responses to West Nile and Insect-Specific Virus Infections in Aedes and Culex Mosquito Cells
Source: Viruses. 2019 Mar 18;11(3):271. doi: 10.3390/v11030271 (PMC6466260; doi:10.3390/v11030271)

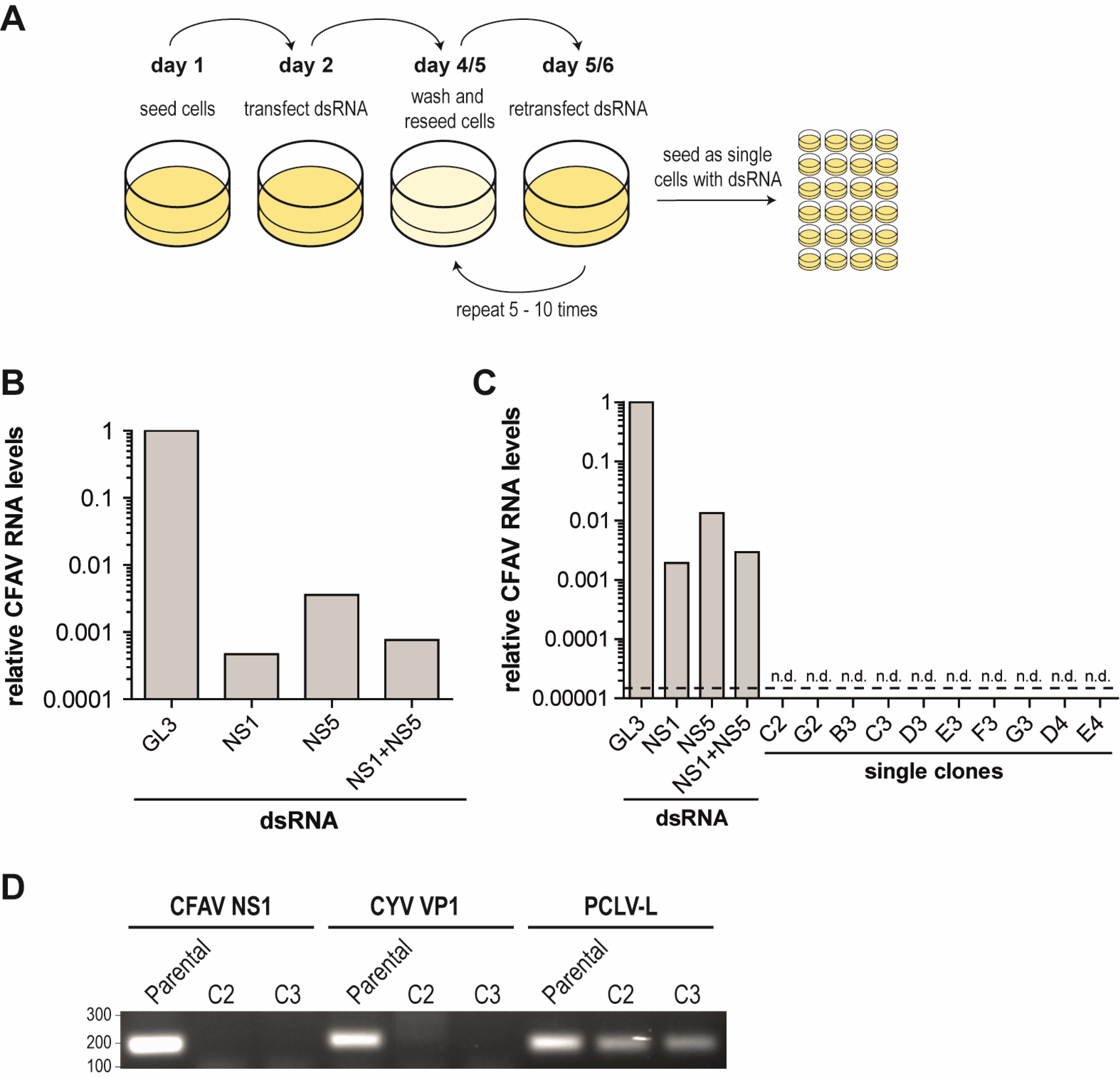

Supplement: Supplementary file 1 [file viruses-11-00271-s001.zip › Supplemental-1.tif]

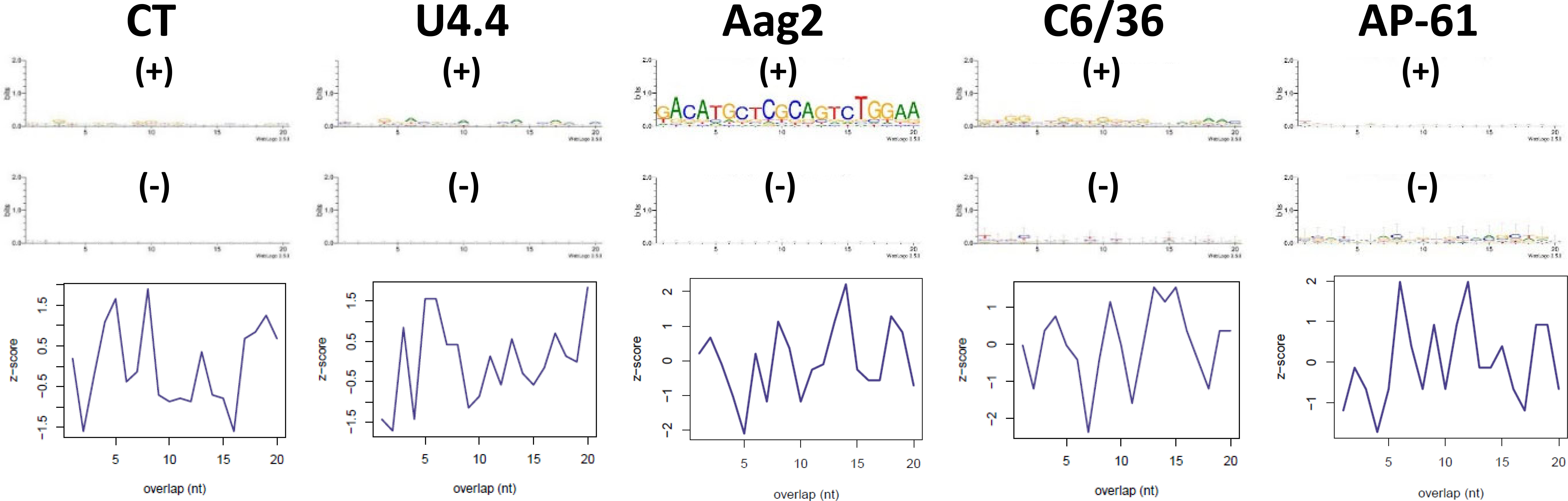

Supplement: Supplementary file 1 [file viruses-11-00271-s001.zip › Supplemental-3.tif]

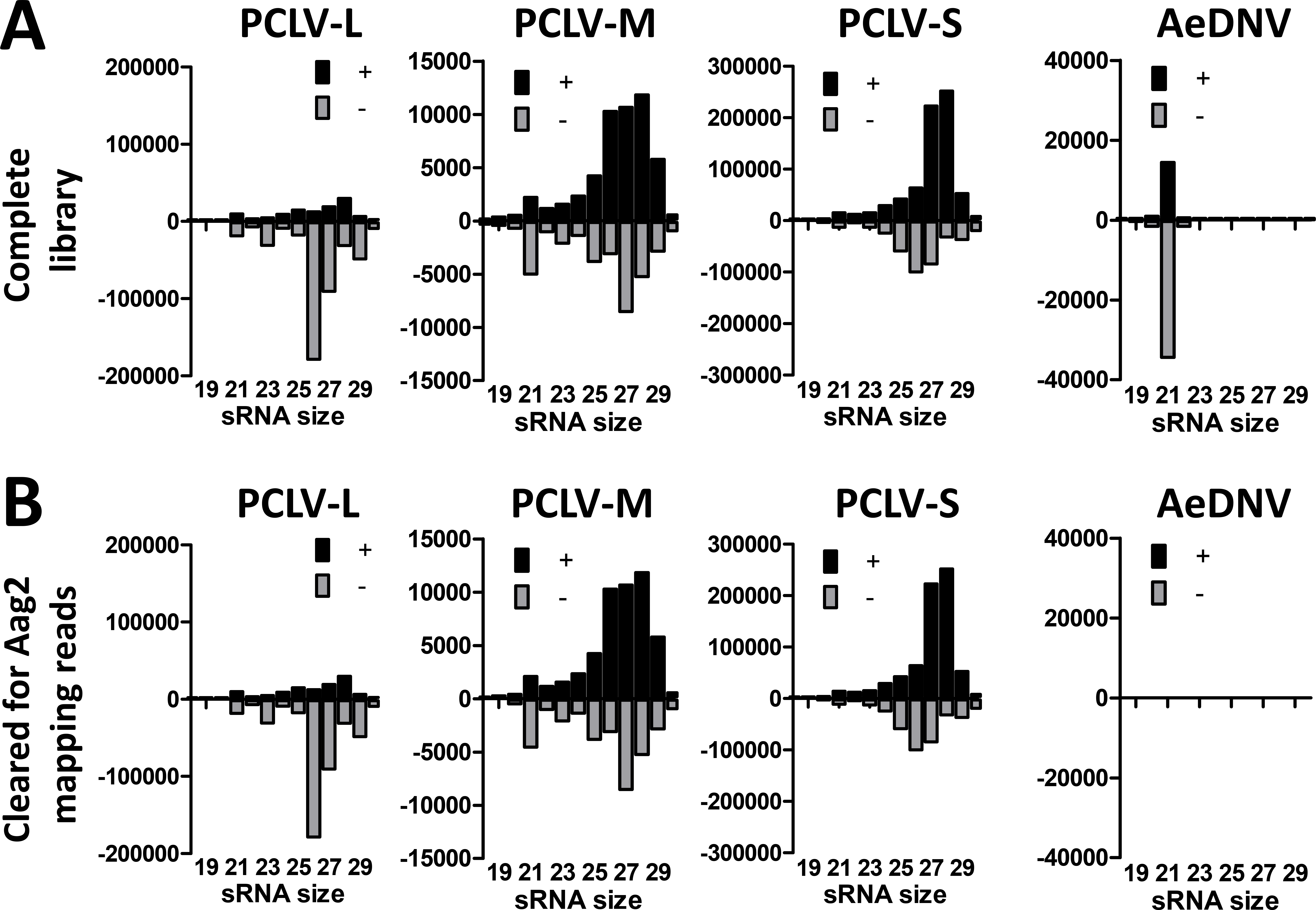

Supplement: Supplementary file 1 [file viruses-11-00271-s001.zip › Supplemental-2.tif]
